# Holidays and Holiday-Makers

**Published:** 1904-08-13

**Authors:** 


### The Hospital.

A Journal of

The Medical Sciences and Hospital Administration.

Vol. XXXVI.—No. 933. AUGUST 13, 1904.

### Holidays and Holiday-Makers.

We lately saw an advertisement of a house which was to be sold or let and which was described by the advertiser as "a good all-the-year-round residence." The description took us back to the days and habits of a long-past childhood when it was the ordinary practice of gentlefolk of moderate means to live all the year round in a single house, and when the more wealthy, who possessed a house in town and a house in the country, were content to divide the year between the two. Even in more recent times we are able to recall the sturdy declaration of a gentleman, well known in official and literary circles, to the effect that he had taken thought and spent money for the purpose of obtaining a perfectly comfortable and healthy habitation of his own, and that he saw no reason why he should imperil the advantages thus gained by sleeping in the houses of other people. All this is changed, and we greatly question whether it be changed for the better. Always by the beginning of August that which in current slang is called the "exodus from London" may be seen to be in full career and the ordinary population of the London streets to be replaced, to a very considerable extent, by tourists armed with cameras, and accompanied by ladies wearing blue veils and carrying crimson covered guide-books. One wonders what the visitors can find to see. The buildings, indeed, remain; so that St. Paul's Cathedral, and Westminster Abbey, and the Houses of Parliament, admit of identification by the curious; but the lingering resident is not surprised to hear exclamations of pained surprise in various tones of the French, the German, and the American languages. "Guess I didn't think there was so little life in London, nohow," is one of the mildest forms of current observation; and the empty parks and the deserted streets and squares completely justify those by whom it is employed.

The disappointments of foreigners may, however, be borne with some semblance of equanimity; and what we are most concerned about are those of our own people. Why should Jones, who has a comfortable house in West Kensington, take the partner of his cares and their six children to some overcrowded so-called "health-resort" (*lucus a non lucendo*), and subject himself and them to a month of bad food, bad cooking, crowded sleeping-rooms, and furniture saturated with the dust of ages. If he would speak the truth he would confess, in nine cases out of ten, that he derives no pleasure from the expedition, and that his only reason for going is because the Smiths are going somewhere, and his wife would not like to remain at home. He tries to escape from deadly boredom by a little extra whiskey and water, and by smoking many more cigars than are good for him; and ultimately returns to his business with nothing to show for his holiday but an embrowned countenance, which will return to its customary pallor in a week.

We are disposed to think that the popular notion of the necessity of holidays is at least greatly exaggerated, and it is certain that it formed no part of the creed of our forefathers. But, admitting, if only for the sake of argument, that people are the better for a period of abstinence from some accustomed kind of work, does it of necessity follow that this period must be spent away from home? London is the healthiest city in the world, its corrected death-rate for 1902 having been only 17·8 per 1,000, against 16·2 for the whole of England and Wales; and it is not doubtful that many of those who leave it for the autumnal exodus do but go farther to fare worse. For those who are able to take a month's rest from their ordinary occupations the resources which London affords are boundless, and are calculated to meet every possible requirement. The treasures of science, of literature, and of art are accessible in limitless profusion. Every study can be pursued, and every accomplishment can be cultivated; yet it is not too much to say that very few Londoners have even a superficial acquaintance with the riches of the great city in which they live. How many are there who could name the most typical forms of architecture presented by its buildings, or the museum or gallery in which some particular branch of study might be most effectively pursued. How many are conversant with the flora and fauna of the home counties, or with the multitudinous wealth of Kew? We would strongly advise some of our readers, when tempted to indulge in a month of vacuous idleness at seaside lodgings, to try as an alternative the effect of a month of intellectual occupation of an unaccustomed kind in London. The brain, like most other bodily organs, may often be relieved from the effects of fatigue by a simple variation in the nature of the demands made upon it; and the great scholars of the Middle Ages, at whose vast attainments our contemporaries can only wonder, mastered the accessible knowledge of their day not by periods of repose, but by seeking rest from mathematics in the pursuit of philology, or by turning from the intricacies of Greek to those of Latin. In these days of quackery, of clamour, and of advertisement, the great requirement of our people is that they should learn to think, and that they should cease from surrendering themselves as a natural prey to every plausible preacher or designing politician who offers to think for them. Would it not be a step in the right direction if we would devote our holidays to the collection of materials upon which thinking could be exercised? Our bodies might, we believe, be trusted to take care of themselves.

